# The therapeutic effect of ozonated olive oil plus glucantime on human cutaneous leishmaniasis

**DOI:** 10.22038/ijbms.2018.29232.7064

**Published:** 2019-01

**Authors:** Maryam Aghaei, Shahrzad Aghaei, Fatemeh Sokhanvari, Nazli Ansari, Sayed Mohsen Hosseini, Mohammad-Ali Mohaghegh, Seyed Hossein Hejazi

**Affiliations:** 1 Skin Diseases and Leishmaniasis Research Centre, Isfahan University of Medical Sciences, Isfahan, Iran; 2 Department of Molecular Medicine, School of Advanced Technologies,Shahrekord University of Medical Sciences, Shahrekord, Iran; 3 Department of Biostatistics & Epidemiology, School of Public Health, Isfahan University of Medical Sciences, Isfahan, Iran; 4 Department of Laboratory Sciences, School of Paramedical Sciences, Torbat Heydariyeh University of Medical Sciences, Torbat Heydariyeh, Iran; 5 Health Sciences Research Center, Torbat Heydariyeh University of Medical Sciences, Torbat Heydariyeh, Iran; 6 Department of Parasitology and Mycology, School of Medicine, Isfahan University of Medical Sciences, Isfahan, Iran

**Keywords:** Amastigote, Antimony compounds Cutaneous leishmaniasis Glucantime, Ozonated olive oil

## Abstract

**Objective(s):**

Leishmaniasis is one of the main health problems in developing countries, caused by intracellular protozoan parasites of the Leishmania genus. Although research has been successful in discovering vaccines and anti-parasitic drugs like antimony compounds, their side effects like high toxicity, prolonged regeneration, etc., have raised the replacement importance of natural products with antioxidant and antibacterial properties. It can be said that an appropriate alternative to this is the ozonated olive oil. Ozone by introducing O_2_ in involved tissues and bloodstream could degrade parasite amastigotes and lead to cleared leishmaniasis infections. So, the present study aimed to evaluate the effect of ozonated olive oil in Iranian leishmaniasis patients compared to glucantime, a choice drug for the treatment of Leishmaniasis.

**Materials and Methods:**

Thirty patients with confirmed leishmaniasis lesions were included and divided into two groups, 15 cases as control and 15 cases as test with lesions of 30–50 mm2 in diameter. The control group received glucantime intralesionally and the test group ozonated olive oil plus glucantime, 2 times daily.

**Results:**

The mean of lesion size was (50.94±33.20) before and (15±14.34) after treatment in control (*P<*0.00) and (50.88±31.74) before and (9.93±14.18) after treatment in the test group (*P<*0.00). Moreover, the mean course of therapy was 10.4(±1.84) weeks and 8.93(±2.15) weeks in control and test groups, respectively (*P=*0.636). Significant differences were reported in lesion size after treatment between the two groups (*P<*0.00).

**Conclusion:**

Data suggested ozonated olive oil can have synergistic effects with glucantime in the treatment of cutaneous leishmaniasis.

## Introduction

Leishmaniasis is a disease caused by a protozoan parasite called *Leishmania*, which is transmitted by female sandfly bites (*Phlebotomus *and *Lutzomyia* genera) (1) and includes cutaneous (2), visceral (3), and mucocutaneous forms (4). The disease has affected more than 12 million people in the world (1). Cutaneous leishmaniasis (CL) is the most common form of leishmaniasis, caused by *Leishmania tropica* and *Leishmania major* (5, 6). Promastigote forms of parasite live in the gut of carrier flies and when they are biting, they get into the injection site and within the phagolysosomes of host macrophages are converted to amastigotes that activate the host cellular immunity response. In the site of the bite, the nodule is caused by the accumulation of lymphocytes, plasma and macrophage cells, and injury caused along with swelling which will last from a few months to years (7). However, cutaneous ulcers without antiparasitic treatment also recover after a few months, the major problem is ulcer duration and scars that remain visible for life after healing. Common drugs (pentavalent antimonials) have side effects such as high toxicity, which led to introduction of new and effective therapies. There are several reports on the healing properties of the herbal oils. There are about 300 known essential oils with anti-viral, fungal, bacterial, and parasitic properties that have commercial importance for pharmacy, agriculture, food, and more. One of the herbal oils is olive oil (virgin). This oil is produced by pressing whole olives, and contains oleic acid (fatty acid with a double bond), vitamin E, and oleuropein (a chemical compound that may affect LDL particle oxidation) (8). A study reported that a mixture of honey, wax, and olive oil could reduce skin inflammation resulting from psoriasis and eczema through the anti-oxidant and anti-inflammatory properties of Oleuropein and prevent the growth of *Candida albicans* and *Staphylococcus aureus* (9). Olive pomace oil is olive oil that is extracted from olive pulp after the first press and is traditionally produced and consumed in Spain. This oil contains large amounts of sterols, tocopherols, waxes, triterpenes, and fatty alcohols (tetracosanol (C24), hexacosanol (C26), octoxane (C28), and doxanel (C22)) with biological activity (10-12). Reducing the secretion of eicosanoid (an inhibitor of the phospholipase A2 activity) by long-chain fatty alcohols suggests that olive oil fatty alcohol may have a protective role against inflammatory agents. They also reduce the production of prostaglandin E2 and TNF-α and significantly decrease the production of thromboxane A2 in peritoneal neutrophils of rats stimulated with calcium A-2318Vionopnore (13). Considering the antimicrobial properties of olive oil, it seems that this compound can be effective in the treatment of leishmaniasis. Ozone therapy is another drug therapy that increases the oxygen amount in the body (14). Ozone is a strong oxidizing agent against gram-positive and negative bacteria, viruses, protozoa, and bacterial and fungi spores (15, 16) that is used in the rapid healing of wounds, immune stimulation, treatment of all cancers and infections, etc. (17). Ozone destroys the bacterial cell coating through the oxidation of phospholipids and lipoproteins and prevents cell growth in certain phases. In viruses, it also damages viral capsids and disrupts reproductive cycles through peroxidation (18). Ozone as a powerful disinfectant for the skin is effective in the removal of protozoan infections such as malaria, giardiasis, trypanosomiasis, and visceral leishmaniasis by secreting oxygen into the bloodstream (19-21). Ozone increases the amount of glycolysis in the red blood cells by stimulating 2-3 diphosphoglycerate, resulting in an increase in the amount of oxygen in the tissue. Ozone also activates the Krebs cycle by increasing oxidative pyruvate carboxylation, which stimulates the production of ATP. Ozone can keep the cell from free radical damage by stimulating the production of superoxide dismutase and catalase and glutathione peroxidase (cell wall enzymes) (22). Ozone can be absorbed slowly in high amounts through the skin and oxidizes toxins to be expelled through the skin rather than stored in the liver. In addition, ozone stimulates the immune system to clear the veins and arteries and reduces inflammation and pain by stimulating the production of hormones and enzymes to normal levels (23, 24). Also, ozone decomposition in inflamed tissues increases oxygen availability, the activity of NFββ transcription factor, and levels of TGF-β2, which leads to the improvement of local metabolism and tissue reproduction and mucosal or cutaneous repair. Therefore, ozone therapy is particularly effective for bed sores, ulcers, burns, and incurable wounds (22, 25). According to reports, ozone can be used in various forms, such as ozonated saline solution (26), ozonated water (27) and ozonated oil (28) and the use of these ointments will be accompanied with destroying the pathogen, reducing inflammation, and rapidly improving the wound (29). Ozone is stabled with bonding in the molecular structure of olive oil and it will have a relatively long half-life of 3 to 6 months by storing ozonized oil at low temperatures (4 ^°^C). Ozonized olive oil retains a small amount of ozone on the skin for a long time and damages the growth of microorganisms, therefore, it can have potential therapeutic effects in preventing secondary infection of surgical wounds, eczema, psoriasis, herpes simplex, acne, etc. (30). Considering the rare research about the therapeutic value of ozonized olive oil on the skin diseases like leishmaniasis, the aim of this study was to evaluate the effect of ozone saturated olive oil on human CL compared with glucantime.

## Materials and Methods

***Study area***

This study was conducted in 2017 in Isfahan, Iran, located in the central part of Iran (latitude 30-34°N and longitude 49-55°E), with moderate and dry weather ranging between 10–40 ^°^C. Isfahan has a population of more than 4 million, which are ethnically Persian/Caucasian. The prevalence of CL caused by *L. major* in this area is significantly greater than many other cities in Iran with about 1315 cases per year, according to a study during 2007–2008.

***Preparation of ozonated olive oil***

In order to provide 150 g of olive oil, about 600–500 cc petroleum solvent and 2 kg pomace olive were combined in a Soxhlet extractor for 3–4 hr. Since the boiling point of oil is higher than petroleum (50 ^°^C), the condenser was used for 2–3 hr to remove the solvent and obtain 100% pure oil. Oxygen gas containing about 200 ppm ozone was then bubbled through the olive oil at a rate of 1.0 l/min for 3 weeks to give an ozonated olive oil with Vaseline consistency and the distinctive odor of ozone. The ozonated olive oil can be kept for 6 months at 4 ^°^C. 

***Study Description***

The statistical population included patients with proven CL who referred to health centers affiliated to Isfahan University of Medical Sciences. After obtaining consent from the Ethics Committee and patients, 30 patients were selected based on entry criteria (aged 20–40, having less than 3 lesions with 1–5 cm^2^ size). Excluded criteria included emerging of complications, having more than 3 lesions, refusal to continue treatment or follow up. Before treatment the area of lesions was measured with a Digimatic caliper (VWRbrand Digital Calipers, Bridgeport, NJ) and the patients were randomly allocated into 2 groups of 15 including test group that was administered ozone saturated olive oil topically for each ulcerative lesion (at the dose of 0.5 ml/mm^2^ twice daily for 8 weeks) along with glucantime (at the dose of 20 mg Sb5+/kg over 20 days) and the control group received a similar volume (20 mg Sb5+/kg) of glucantime, a choice drug for the treatment of Leishmaniasis. Patients were interviewed weekly during therapy for dermatological side effects (e.g., pain, erythema, and edema), and the size of each lesion (mm^2^) in 2 dimensions (length and width) was measured with digital calipers weekly with a two-month forecast for the treatment. After the completion of the treatment period, patients in the test and control groups were followed for 6 months. Lesion cure was defined as 100% reepithelialization of the lesion without relapse in the 6-month follow-up. Determination of lesion cure and failure was made by a clinician blinded to the treatment group of the patients. The protocol’s endpoint was the cure of the patient with all of their lesions resolved. Ultimately, the therapeutic effect of ozonated olive oil was assessed by comparing the skin lesion size of test and control groups.

***Statistical analysis***

The data were analyzed by mean±SEM (standard error of the mean). The statistical analysis of the differences in lesion size was carried out by using repeated measure and t-test in different treatment groups. All statistical analyses were performed in SPSS version 16. Values of *P*<0.05 were statistically considered significant.

## Results

***Patient characteristics***

In this study, among 30 patients, the test group included 15 patients (5 females and 10 males, F: M =1:2) and 15 cases were randomized to the control group (4 females and 11 males, F:M=1:2.75). Mean age was 35.53 (±13.22) years in the test group and 34.26 (±13.80) years in the control group. The mean number of lesions was 2 per patient, as 26.7% of the patients suffered a unique lesion and 66.7% presented 2–3 lesions. Patient lesions presented on their upper limbs (45.3%), as lesions located mainly on the face and hands (33.3%), lesions on legs (7.7%), and rarely the thoracic-abdominal region (4.7%). The lesion types included ulcerative lesions and non-ulcerative (nodular and plaques). All patients received at least one complete round of glucantime (20 mgSb5+/kg/day over 20 days) and only 12.8% and 3,9% of patients had two or three complete cycles, respectively. The most common side effects after therapy with glucantime were: arthralgia (39.7%), headache (28.2%), fever (26.3%), myalgia (23.5%), nausea (8.6%), diarrhea (10.2%), and other symptoms such as facial swelling (15.87%). Except for a transient burning sensation in some of the patients, the treatment with ozonated olive oil was without adverse effects and was well tolerated. Although the mean duration of therapy was 10.4(±1.84) weeks at control versus 8.93(±2.15) weeks in the test group (*P*<0.668), cure rate in the group treated with ozonated olive oil and glucantime was higher than in the group receiving glucantime only. Also, there was a significant relationship between duration of treatment and number of lesions, as longer treatment was required for reducing the area or causing small lesions to disappear completely. Cure rate in both groups was correlated with age and location of lesions, as younger patients and lesions located in hands or face had a rapid rate of healing. 

Baseline data of all subjects (test and control) are shown in Table 1.

***The lesion size measurements***

**Figure 1:**
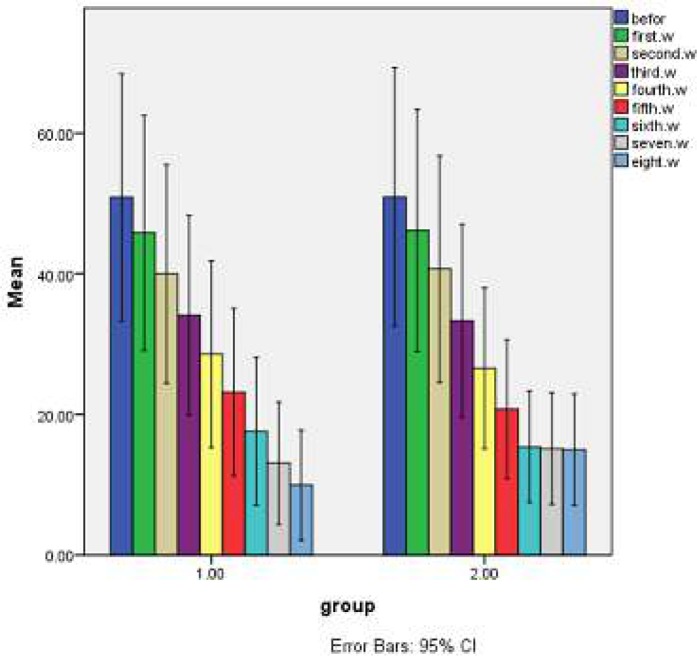
The mean of lesions size (mm^2^) in control (1) and test (2) groups weekly

Lesion size measurements at baseline and weekly, up to eight weeks were done. The results showed a significant decrease in the size of lesions in the control group in different weeks, while the difference was more frequent and continuous in the test group, as the lesion size reduced in up to six weeks but did not change in the control group in seven and eight weeks, while it decreased in the test group in the last two weeks. This difference could have resulted from the effect of ozonated olive oil. Therapeutic effects of ozonated olive oil and glucantime on the lesion sizes (mm^2^) of test and control groups were described weekly in Table 2, Figure 1a, and Figure 1b. 

**Figure 2:**
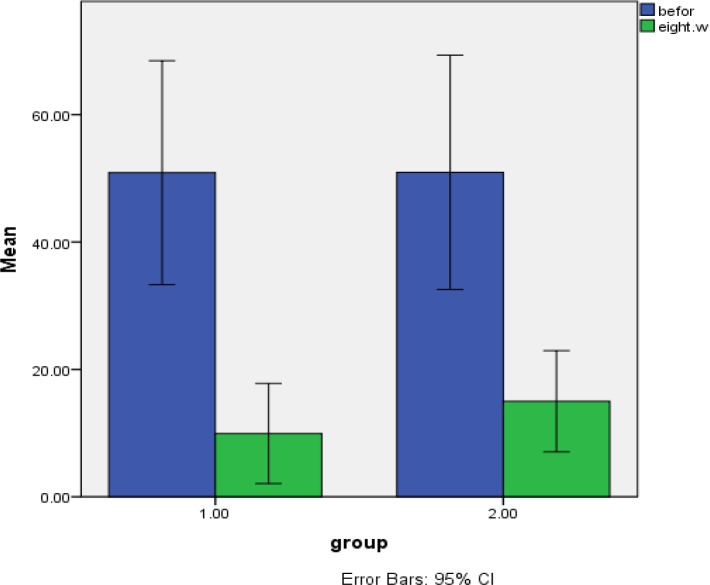
Lesion size means at baseline and during 8 weeks in test group receiving ozonated olive oil and glucantime (1) and the control group that received glucantime alone (2)

An obvious difference was found in lesion size 8 weeks after treatment between the two groups (*P*<0.00). Also, a significant reduction in lesion size was observed during 8 weeks in comparison with before treatment in both groups (*P*<0.000) (Figure 2). 

**Figure 3:**
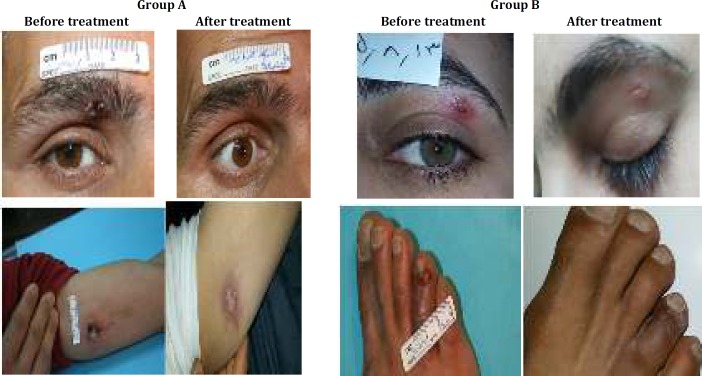
The size of CL lesion. Group A: test group treated with ozonated olive oil and glucantime. Group B: control group treated with glucantime alone

Also, our work showed that administration of ozonated olive oil topically reduced the severity of the ensuing lesion significantly. As healing was excellent and all lesions flattened considerably and most lesions left only superficial scarring or slight post inflammatory hyperpigmentation. These changes were shown in Figure 3. 

## Discussion

*L. major* is a causative agent of CL in many parts of Iran. Since antileishmanial chemotherapy being used for leishmaniasis has limitations like high cost, management difficulty, and high toxicity and resistance, therefore, there is an urgent need for designing a new, safe, more effective and economically feasible drug for the treatment of CL. In this regards, plant extractions such as olive oil with high killing effects on *Leishmania* parasites and low cytotoxicity for human cells are favorable and promising candidates for CL treatment (1, 8). Kyriazis and colleagues in 2013, investigated the anti-amastigote and promastigote activity of oleuropein and hydroxytyrosol in olive leaf and olive-mill waste and showed that both have inhibitory effects in the logarithmic and stationary phases of *L. infantum*, *L. donovani,* and *L. major* promastigotes. Also, oleuropein has shown an anti-*Leishmania* effect in a mice model as the parasite burden of the spleen decreased, and this effect was sustained even after 6 weeks of follow up (31). Another study in 2014, revealed oleuropein’s nontoxicity and apoptosis and anti-proliferative effects on *Leishmania* promastigotes in comparison with pentastom (32).

 Also, ozone has been shown to have powerful and reliable therapeutic effects against bacteria, fungi, protozoa, and viruses, as the oxidant potential of ozone induces the destruction of the cell walls and cytoplasmic membranes of bacteria and fungi. During this process, ozone attacks glycoproteins, glycolipids, and other amino acids and inhibits and blocks the enzymatic control system of the cell. The degradation of nucleic acid was parallel to what happened to enzymatic activities. This leads to an increase in membrane permeability that plays a key role in cell viability, leading to prompt functional cessation. It lets ozone molecules penetrate the cell resulting in microorganism death (24-27). In one study in 2015, researchers worked out ozone therapy for radiation reactions and skin lesions after neutron therapy in patients with malignant tumors and showed ozone therapy safety and increased efficiency of complex treatment of these patients (33). Furthermore, in 2016, Rosul and Patskan reported the positive effect of ozone therapy along with surgical treatment among 47 patients with stages I and II of diabetic foot in terms of the treatment duration and wound process, promoting the improvement of lipid peroxidation and antioxidant protection indexes (34).

**Table1:** Baseline data of test and control groups

	** Test** ** (n=15)**	**Control (n=15)**	***P*** **-value**
**Age (years)**	35.53 (±13.22)	34.26 (±13.80)	0.852
**Mean duration of treatment (week)**	8.93(±2.15)	10.4(±1.84)	0.636
**Mean number of lesions (±SD)**	2.13(±0.83)	2.06(±0.79)	0.668
**Mean lesion location ** (upper limbs)	5.26(±3.76)	4.86(±3.88)	0.815

**Table 2:** Frequency distribution ± SD of the average size of lesions in different groups (mm^2^).

**Week**	**Test Group**	**Control Group**
**Before treatment**	50.88±31.74	50.94±33.20
**First week**	45.78±30.19	46.16±31.15
**Second week**	39.98±28.08	40.66±29.12
**Third week**	34.10±25.63	33.33±24.77
**Fourth week**	28.58±23.94	26.54±20.66
**Fifth week**	23.15±21.54	20.76±17.85
**Sixth week**	17.61±19.02	15.38±14.31
**Seventh week**	13.05±15.82	15.14±14.35
**Eight week**	9.93±14.18	15±14.34

Although ozone therapy requires the gaseous form to be more effective, it has been proposed that the base oils such as olive oil, during the ozonation processes can engage O_3_ in the form of a stable ozonide (35). The reaction of ozone with olive oil occurs almost exclusively with c-c double bonds present in unsaturated fatty acids and when this ozonide encounters the warm exudates of the wound, they slowly disintegrate to reactive ozone and readily dissolve in water and produce several oxygenated compounds such as hydroperoxides, polyperoxides, aldehydes, ozonide, and diperoxides that have antimicrobial and stimulatory activities on ulcerated chronic wounds (36). Also, ozone therapy with changing cell types and release of cytokines modulate the complex healing process (35). Since there is no study on anti-leishmanial activity of ozonated olive oil in humans, we studied dual therapy of ozonated olive oil and glucantime on leishmaniasis patients and obtained results showed distinct improvement of the disease. So that the wound sizes treated with ozonated olive oil and glucantime were decreased considerably (from 50.88 mm^2^ to 9.93 mm^2^) in comparison with wounds treated with glucantime alone (size from 50.94 mm^2^ to 15 mm^2^) with a mean difference of 5.07 mm^2^. In agreement with our results, Rajabi *et. al.* obtained positive biological effects with ozone saturated olive oil in *L. major* promastigotes and infected mice, as the mortality rate of parasites increased significantly after using oil. Also, the use of this substance in mice with CL ulcers led to reduced wound size in comparison with the control group. Moreover, Rajabi *et. al*. showed significant differences in parasite survival percentage between ozonated olive oil and non-ozonated olive oil, at similar concentrations (*P*<0.001) and the leishmanicidal effect of ozonated olive oil was more effective than glucantime (36). Furthermore, the therapeutic effect of ozonated olive oil has been investigated on other ulcers. For example, researchers in 2009 assayed therapeutic effects of topical application of ozone on acute cutaneous wounds of sixteen female guinea pigs. Their results showed that the ozonated olive oil significantly enhances the healing effects on acute cutaneous wounds as compared to the oil as well as the control groups. As on day 11, all of the wounds completely reepithelized irrespective of treatments. The ozone group showed a significantly smaller wound size than the oil group on days 5 (*P*<0.05) and 7 (*P*<0.01) (37). At 2013, a study used topical ozonated oil on an exophytic enlarged gingival lesion, and the therapeutic benefits were obtained after one-week. The morphometrical analysis revealed a reduction in the mean area (from 108.64 mm^2^ to 96.35 mm^2^) of the gingival lesion after the application of ozonated oil (38). In 2016, the efficacy of ozonized olive oil was evaluated in the treatment of oral lesions and conditions of 50 patients (aphthous ulcerations, herpes labialis, oral candidiasis, oral lichen planus, and angular cheilitis). Improvement in the signs and regression of lesions were seen in all patients (39).

## Conclusion

The good effects of ozonated olive oil in this study suggest that ozone therapy can be used either in monotherapy or in combination with other drugs for repairing lesions of leishmaniasis and even other ulcers. Further studies are required to investigate the effect of different concentrations of ozonated olive oil for treating leishmaniasis lesions.
